# Sodium-glucose Cotransporter 2 Inhibitors and Pathological Myocardial Hypertrophy

**DOI:** 10.2174/1389450124666230907115831

**Published:** 2023-11-16

**Authors:** Zhicheng Gao, Jiaqi Bao, Yilan Hu, Junjie Tu, Lifang Ye, Lihong Wang

**Affiliations:** 1 The Second Clinical Medical College, Zhejiang Chinese Medical University, Hangzhou, People’s Republic of China;; 2 Heart Center, Department of Cardiovascular Medicine, Zhejiang Provincial People’s Hospital (Affiliated People’s Hospital), Hangzhou Medical College, Hangzhou, Zhejiang, China

**Keywords:** Sodium-glucose cotransporter 2 inhibitors, myocardial hypertrophy, myocardial fibrosis, inflammation, epicardial adipose tissue, Na^+^/H^+^ exchanger, insulin resistance, autophagy

## Abstract

Sodium-glucose cotransporter 2 (SGLT2) inhibitors are a new type of oral hypoglycemic drugs that exert a hypoglycemic effect by blocking the reabsorption of glucose in the proximal renal tubules, thus promoting the excretion of glucose from urine. Their hypoglycemic effect is not dependent on insulin. Increasing data shows that SGLT2 inhibitors improve cardiovascular outcomes in patients with type 2 diabetes. Previous studies have demonstrated that SGLT2 inhibitors can reduce pathological myocardial hypertrophy with or without diabetes, but the exact mechanism remains to be elucidated. To clarify the relationship between SGLT2 inhibitors and pathological myocardial hypertrophy, with a view to providing a reference for the future treatment thereof, this study reviewed the possible mechanisms of SGLT2 inhibitors in attenuating pathological myocardial hypertrophy. We focused specifically on the mechanisms in terms of inflammation, oxidative stress, myocardial fibrosis, mitochondrial function, epicardial lipids, endothelial function, insulin resistance, cardiac hydrogen and sodium exchange, and autophagy.

## 1 INTRODUCTION

**Fig. (1):**
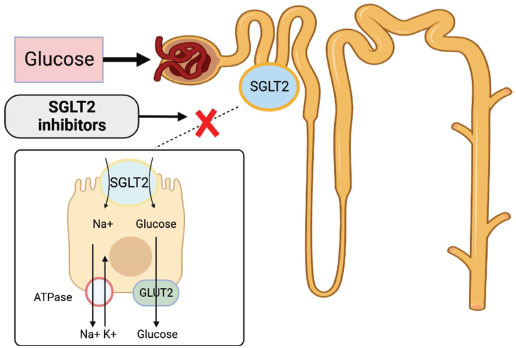
Mechanism of SGLT2 inhibitors in the renal tubules.

Sodium-glucose cotransport protein 2 (SGLT2) inhibitors are a new type of oral hypoglycemic agents used to treat type 2 diabetes mellitus. SGLT2, mainly located in the kidney, is a glucose transporter protein responsible for transporting glucose from the tubular lumen to the tubular epithelium and reabsorbing approximately 90% of the glucose filtered by the kidney. SGLT2 inhibitors exert their hypoglycemic effects mainly by blocking the reabsorption of glucose in the proximal renal tubules and promoting glucose excretion (Fig. **1**) [1, 2]. This type of glucose-lowering is not insulin-dependent and is accompanied by a very low risk of hypoglycemia. SGLT2 inhibitors can also lower blood pressure, glycated hemoglobin, and body weight [3].

Pathological myocardial hypertrophy is related to myocardial fibrosis and structural remodeling [4], which usually increases the risk of adverse cardiovascular events [5]. In addition to the common causes of increased cardiac afterload, such as hypertension and aortic stenosis, diabetes can also specifically cause pathological myocardial hypertrophy in patients through cardiac microvascular disease, myocardial metabolic disorder, and myocardial fibrosis [6]. In fact, left ventricular hypertrophy, which occurs in approximately 40% to 70% of patients with type 2 diabetes mellitus (T2DM), is independently associated with cardiovascular events and even causes more deaths than multivessel coronary artery disease [7]. Moreover, this cardiovascular risk will decrease with the regression of left ventricular hypertrophy and even return to normal after complete regression of left ventricular hypertrophy [8].

Although SGLT2 inhibitors are predominantly expressed in the kidney, direct cardioprotective effects of SGLT2 inhibitors have been demonstrated. It was recently found that the cardiovascular outcomes of T2DM have been improved due to the utilization of SGLT2 inhibitors, especially in the prevention of hospitalization for heart failure [9-11]. Pabel *et al.* summarized the different multidirectional mechanisms of SGLT2 inhibitors in improving the state of heart failure in their review, including the effects on ventricular remodeling [12].

Recently, different reports suggest that SGLT2i may be involved in ventricular remodelling. The EMPA-HEART Cardiolink-6 trial found that the left ventricular mass, measured by body surface area, decreased significantly after 6 months of EMPA treatment in patients with T2DM and coronary artery disease [13]. Consistent with the trial mentioned above, Brown *et al.* [14] proposed a randomized controlled experiment (DAPA-LVH). They further demonstrated that the SGLT2 inhibitor, dapagliflozin, plays a crucial role in the regression of left ventricular hypertrophy in patients with T2DM and can lead to reverse remodeling and changes of left ventricular structure [14].

In animal studies, the SGLT2 inhibitor, empagliflozin (EMPA), has been shown to have protective effects on myocardial hypertrophy and diastolic function in mice with prediabetic metabolic syndrome [15]. Myocardial hypertrophy and myocardial fibrosis induced by chronic cortisol in mice were improved after 4 weeks of EMPA treatment [16]. Moreover, a decrease in left ventricular mass and septal thickness and improvement in cardiomyocyte hypertrophy and ventricular fibrosis were found in rats with non-diabetic cardiomyopathy treated with the SGLT2 inhibitor, ipragliflozin, without altering blood glucose levels [17].

Increasing evidence has shown that cardiomyocyte hypertrophy and myocardial fibrosis are improved after the application of SGLT2 inhibitors, whether accompanied by diabetes or not (Table **1**). The improvement of myocardial hypertrophy and its beneficial effect on ventricular remodeling may be the core mechanism of the beneficial cardiac effects of SGLT2 inhibitors [12]. However, the potential mechanisms by which SGLT2 inhibitors attenuate pathological myocardial hypertrophy remain to be elucidated. Therefore, we reviewed the available mechanisms of SGLT2 inhibitors to attenuate pathological myocardial hypertrophy with the aim of providing a reference for the future treatment thereof.

## 2 MECHANISM INSIGHTS

Diabetes causes a decrease in the number of cardiomyocytes and promotes cardiomyocyte hypertrophy, which may be caused by the activation of apoptotic mechanisms. It also causes a decrease in proliferative potential; in either case, the hypertrophy may be compensatory [18]. The renin-angiotensin-aldosterone system is activated by hyperglycemia, which increases the expression level of angiotensin II (Ang II) [19], which further stimulates the proliferation of cardiac fibroblasts, induces cardiomyocyte hypertrophy, and finally leads to myocardial hypertrophy [20].

Hyperglycemia also induces the activation of protein kinase C (PKC); notably, the PKCβ2 isoform is preferentially overexpressed, which induces cardiomyocyte hypertrophy by damaging caveolin-3 expression and insulin metabolism of Protein Kinase B (Akt)/endothelial nitric oxide synthase signaling [21]. In addition, hyperglycemia directly reduces parasympathetic activity, resulting in cardiac autonomic neuropathy with relatively high sympathetic nervous system activity [22, 23]. In contrast, elevated sympathetic nervous system activity promotes β-1 adrenergic receptor signaling and induces myocardial cell hypertrophy and interstitial fibrosis [24]. SGLT2 inhibitors were originally developed as a hypoglycemic agent that inhibits renal tubular reabsorption of glucose to lower blood glucose by increasing glycosuria, an insulin-independent mechanism that appears to provide durable hypoglycemic efficacy at any stage of the natural course of T2DM [25], thereby reducing pathological myocardial hypertrophy.

**Fig. (2):**
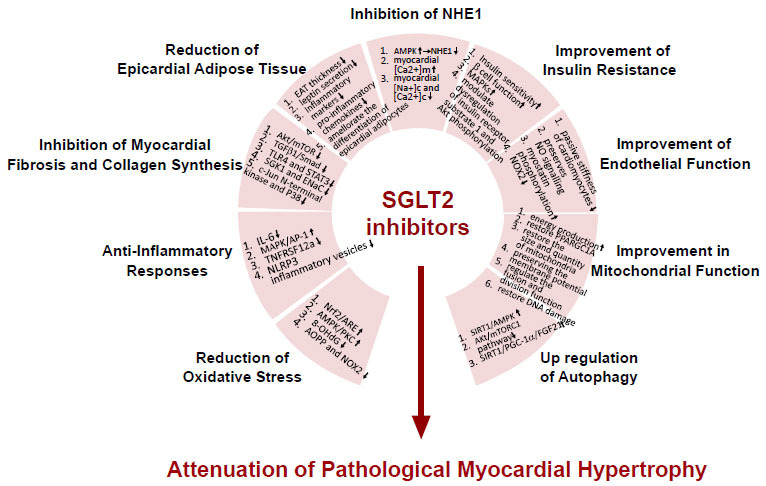
Mechanisms of SGLT2 inhibitors in attenuating pathological myocardial hypertrophy.

There is growing evidence that cardiomyocyte hypertrophy and myocardial fibrosis are ameliorated with the application of SGLT2 inhibitors, even without T2DM. In these studies, many mechanisms by which SGLT2 inhibitors attenuated pathological myocardial hypertrophy were observed that were independent of the hypoglycemic effect (Fig. **2**).

## 3 REDUCTION OF OXIDATIVE STRESS

A few previous research studies have demonstrated that oxidative stress plays a significant part in myocardial hypertrophy and remodeling [26-28]. Oxidative stress is defined as the overproduction of reactive oxygen species (ROS), which leads to cellular damage by damaging deoxyribonucleic acid (DNA) and proteins [29].

Multiple research studies have suggested that ROS has a vital impact on myocardial hypertrophy induced by a high salt diet [30, 31] and causes myocardial structural impairment and systolic dysfunction [32]. A previous study on isolated cardiomyocytes showed that a tiny augment in ROS leads to a phenotype characteristic of hypertrophy, which is of great importance to cardiac remodeling [33].

Nuclear factor-like erythroid factor 2 expresses antioxidant and cytoprotective genes by interfering with antioxidant response elements [34]. Taking the T2DM KK-Ay mouse as a model, Li *et al.* [35] found that EMPA could inhibit cardiac oxidative stress through the activation of nuclear factor-like erythroid factor 2/antioxidant response elements signaling, thereby reducing myocardial hypertrophy.

Adenosine-monophosphate-activated protein kinase (AMPK) is a cellular energy sensor that is activated to augment the production of adenosine triphosphate (ATP) and reduce the consumption of ATP [36]. In previous studies, AMPK inhibited the phosphorylation of PKC to suppress ROS [37]. Furthermore, several research studies have reported that dapagliflozin may alleviate oxidative stress by the AMPK/PKC signaling pathway [38, 39].

Yurista *et al.* [40] also demonstrated that EMPA attenuated total myocardial oxidative stress, as evidenced by a reduction in advanced oxidation protein product and superoxide-generating enzyme nicotinamide adenine dinucleotide phosphate oxidase-2 expression.

In addition, 8-Oxo-2'-deoxyguanosine is an appropriate biomarker for evaluating oxidative stress [41]. The selective SGLT2 inhibitor, TA-1887, attenuates oxidative stress and the advantages are reflected in a reduction in 8-Oxo-2'-deoxyguanosine levels [42].

## 4 ANTI-INFLAMMATORY RESPONSES

Transverse aortic constriction (TAC) is a commonly used way of leading to an acute pressure overload model. Evidence suggests that TAC induces an inflammatory response and stimulates cardiac hypertrophy [43]. One of the regulatory molecular pathways in charge of the mediation of cardiac hypertrophy is the tumor necrosis factor receptor superfamily member (TNFSF12)/ tumor necrosis factor receptor superfamily member 12a (TNFRSF12a) system [44]. Yerra *et al.* [45] discovered that the expression of TNFRSF12a in cardiomyocytes was significantly up-regulated after TAC operation. Under the induction of mechanical strain, TNFRSF12a exerts its feedforward function, activates the signal cascade promoting hypertrophy and inflammation, and enhances pathological cardiac remodeling, presenting as ventricular hypertrophy and decreased cardiac function [46-49]. In addition, the role of TNFSF12/TNFRSF12a as a positive regulatory factor in cardiac hypertrophy was verified in the experimental model of right ventricular hypertrophy [47].

Inflammation exerts an enormous influence on the evolution of myocardial hypertrophy [50, 51]. For example, interleukin (IL)-6 has been proven to be related to cardiac hypertrophy [52]. Elevated free fatty acids, for example, palmitic acid, are thought to be associated with obesity-induced left ventricular hypertrophy [53], and free fatty acids can directly activate some harmful pathways, induce inflammatory responses, and promote cardiomyocyte hypertrophy and apoptosis [54, 55]. In addition, nucleotide oligomerization domain-like receptor pyrin domain containing 3 (NLRP3) inflammatory corpuscles play an important part in S-nitrosylation of muscle LIM protein-induced myocardial hypertrophy as a downstream signaling pathway [56].

A recent animal study found that the SGLT2 inhibitor, ertugliflozin, modulates inflammatory processes to attenuate pressure overload-caused myocardial hypertrophy and adverse cardiac reconstitution [57]. Additionally, EMPA reduces the upregulation of TNFRSF12a in the hearts of mice undergoing TAC operation, thereby alleviating myocardial cell hypertrophy [45]. In addition, canagliflozin, another SGLT2 inhibitor, has been proven to lower serum IL-6 levels [58]. EMPA also has anti-inflammatory properties and reduces inflammatory responses by lowering myocardial IL-6 [59].

Lin *et al.* [60] demonstrated that dapagliflozin pretreatment alleviated palmitic acid-mediated cell hypertrophy in a concentration-dependent manner. This protective effect was achieved by dapagliflozin through the suppression of sodium-hydrogen exchanger isoform-1 (NHE1)/mitogen-activated protein kinase (MAPK)/ activator protein 1 (AP-1) pathway-mediated inflammation in cardiomyocytes. Interestingly, a previous animal study demonstrated that inhibition of SGLT2 by dapagliflozin mediated the attenuation of NLRP3 inflammatory vesicles [61]. Recently, a similar conclusion was reached in a rodent model: EMPA blunts NLRP3 inflammatory vesicle activation in a Ca^2+^-dependent manner, thereby reducing cardiac inflammation [62]. In addition, in patients with T2DM, EMPA also attenuated the activation of NLRP3 inflammatory vesicles by elevating serum β-hydroxybutyrate and lowering serum insulin [63].

In summary, SGLT2 inhibitors promote these anti-inflammatory processes, thereby reducing cardiac hypertrophy caused by various inflammatory pathways and exerting a cardioprotective effect.

## 5 INHIBITION OF MYOCARDIAL FIBROSIS AND COLLAGEN SYNTHESIS

Pathological myocardial hypertrophy is associated with myocardial fibrosis as well as cardiac structural remodeling [4]. Myocardial fibrosis can result in the development of left ventricular reconstitution and diastolic dysfunction [64]. The potential pathological and initiating elem mediators contribute to the evolution of myocardial fibrosis.

Ang II is an effective activator of cardiac fibrosis and myocardial cell hypertrophy [65]. In a wide range of fibrotic signaling pathways, transforming growth factor-β1 (TGFβ1)/Smad is essential for inducing and maintaining collagen synthesis and the activation of cardiac fibroblasts, and it mediates structural remodeling caused by Ang II to a certain extent [66]. Ang II-induced cardiac reconstruction is also dependent on the phosphorylation of signal transducer and activator of transcription 3 (STAT3) through toll-like receptor 4 (TLR4), and TLR4 knockdown significantly attenuates Ang II-induced STAT3 activation [67]. Matsuda *et al.* [68] elucidated that TLR4 deficiency improved Ang-II-mediated cardiomyocyte hypertrophy as well as myocardial fibrosis.

In an experiment regarding human cardiac fibroblasts, EMPA has been proven to alleviate TGFβ1-mediated fibroblast activation as well as cell-mediated collagen remodeling [69]. Further studies revealed that dapagliflozin could modulate TGFβ1/Smad signaling in a glycemic-independent manner and attenuate Ang II-induced myocardial fibrosis and collagen synthesis [70]. A recent animal study found that corticosterone treatment activated myocardial STAT3 and upregulated TLR4 expression in mice, resulting in myocardial hypertrophy and fibrosis. In contrast, EMPA treatment alleviated myocardial hypertrophy and fibrosis by down-regulating TLR4 expression and inhibiting STAT3 phosphorylation [16].

Serum- and glucocorticoid-responsive kinase-1 (SGK1) is a serine-threonine kinase expressed in large quantities in the heart [71]. SGK1 modulates the expression of many ion channels containing epithelial sodium channels [72]. Increasingly, studies have confirmed that SGK1 and epithelial sodium channels have a contributing effect on fibrosis and harmful myocardial hypertrophy [71, 73]. SGK1 was activated and induced ventricular remodeling, including fibrosis and ventricular hypertrophy, after aorta fasciculation in rats [71, 73].

Habibi *et al.* [74] discovered that EMPA can decrease the expression of SGK1 and epithelial sodium channels protein in the myocardium and normalize their level, thus alleviating myocardial fibrosis.

The mammalian target of rapamycin (mTOR) is a major modulator of cell growth and protein synthesis [75]. Cardiac mTOR signaling plays a key role in the evolution of the organ [76]. However, sustained mal-regulated activation of mTOR during heart failure promotes ventricular hypertrophy as well as detrimental cardiac remodeling; however, partial inhibition of mTOR prevents ventricular fibrosis in hypertrophic cardiomyopathy models [75, 76]. Akt is a serine/threonine protein kinase and participates in hypertrophic cardiomyopathy through the mTOR pathway. Inhibiting Akt signaling can ameliorate maladaptive hypertrophy and fibrosis [77].

Moellmann *et al.* [57] found that 10 weeks of ertugliflozin treatment can reduce the mTOR associated with cardiomyocyte hypertrophy and detrimental cardiac remodeling. In addition, inhibition of SGLT2 produces massive glycosuria, causing a large loss of calories *via* the urine, which triggers a fasting-like transcriptional pattern [78, 79]. Interestingly, the SGLT2 inhibitors-induced fasting-like transcriptional pattern may inhibit the Akt/mTOR [79] pathway, thus improving maladaptive hypertrophy and fibrosis. A recent animal study found that EMPA inhibited Akt/mTOR phosphorylation, which may exert an effect in ameliorating hypertrophy and fibrosis [80].

The MAPK pathway plays a crucial part in myocardial remodeling as well, especially during the process of fibrosis [81]. A research study has demonstrated that dapagliflozin could prevent cardiac remodeling through the inhibition of c-Jun N-terminal kinase and P38, two main proteins of the MAPK pathway [82].

In summary, SGLT2 inhibitors protect myocardial hypertrophy and fibrosis caused by various mediators and signaling pathways.

## 6 REDUCTION OF EPICARDIAL ADIPOSE TISSUE

Epicardial adipose tissue (EAT) is a particular fat depot located in the heart and is the body’s visceral fat. Healthy EAT secretes cardioprotective adipokines that provide nutrients for the heart muscle, such as adiponectin, an adaptive adipokine derived from adipocytes that can protect the hypertrophic stimulation of cardiomyocytes as well as the inflammation and fibrosis of the myocardium [83]. Nevertheless, while affected by systemic inflammation or metabolic disorder, the epicardium may become the site of adipogenesis disorder. For example, diabetes can cause the expansion of epicardium adipose tissue, contributing to the synthesis of several proinflammatory adipocytokines, such as leptin, TNF-a, and IL-6. As there is no myofascia to separate the myocardium from EAT, the two tissues share the same microcirculation, which allows pro-inflammatory adipocytokines synthesized in epicardial fat tissue to be secreted directly into the adjacent myocardium, leading to myocardial inflammation and fibrosis [83]. This also explains why the volume of EAT is strongly correlated with cardiac fibrosis as well as left ventricular hypertrophy [84, 85].

A previous 24-week randomized controlled experiment demonstrated that EMPA rapidly and significantly reduced EAT thickness [86]. Canagliflozin has the same effect [87]. Subjects who received treatment with ipragliflozin and another SGLT2 inhibitor, luseogliflozin, had reduced volume of EAT as well [88, 89]. Recently, EMPA was related to a significant decrease in EAT in non-diabetic patients with heart failure [90]. Furthermore, SGLT2 is expressed in human EAT. Inhibition of SGLT2 by dapagliflozin can also ameliorate the differentiation of epicardial adipocytes and decrease the production of EAT pro-inflammatory chemokines and inflammatory markers [91, 92].

Leptin is a protein derived mainly from adipose tissue [93]. The expansion of EAT can promote its synthesis, and circulating leptin levels correlate with epicardial fat mass [94], although the precise mechanisms remain unclear. However, experiments have shown that leptin can directly lead to cellular hypertrophy in rodent and human myocardial cells [93, 95, 96].

Leptin also promotes adverse changes in ventricular geometry and stimulates collagen synthesis, which can lead to cardiac fibrosis [97].

Based on the above benefits of SGLT2 inhibitors on the quality and biology of EAT, it is hypothesized that these agents may exert an antagonistic effect on leptin [98]. The available experimental data demonstrated that inhibition of SGLT2 further inhibits leptin secretion from adipocytes [99]. Further clinical trials found that SGLT2 inhibitor treatment reduced circulating leptin levels in patients undergoing insulin-resistant states and that this effect was disproportionate to weight loss [100-102].

## 7 IMPROVEMENT IN MITOCHONDRIAL FUNCTION

It is well known that cardiomyocytes are mainly powered by ATP produced by mitochondrial respiration [103], and both basic experiments and clinical research have demonstrated that mitochondrial dysfunction results in myocardial hypertrophy [104]. Impaired mitochondrial function leads to increased extracellular matrix, which causes myocardial contractile dysfunction, leading to myocardial fibrosis and hypertrophy [105].

EMPA has been proven to improve cardiac mitochondrial function [106]. Elevated intracellular sodium levels may impair mitochondrial calcium handling, leading to mitochondrial dysfunction [107]. Interestingly, EMPA can reduce sodium overload in cardiomyocytes by inhibiting sodium-hydrogen exchangers while elevating mitochondrial calcium [108], thereby activating mitochondrial dehydrogenase to accelerate substrate oxidation, increasing mitochondrial energy production, and improving mitochondrial dysfunction [109]. This improves myocardial contractile function and reduces myocardial hypertrophy.

EMPA provides a direct protective effect against hyperglycemia-induced mitochondrial damage as well [110]. Peroxisome proliferator-activated receptor-gamma coactivator-α (PPARGC1A) is the key mediator in mitochondrial biogenesis; thus, a decrease thereof is thought to contribute to mitochondrial dysfunction. Animal experiments revealed that EMPA treatment restored mitochondrial function by restoring PPARGC1A in the hearts of prediabetic rats [111]. Mizuno *et al.* [107] confirmed that EMPA restored the size and quantity of mitochondria in hearts after myocardial infarction. In addition to preserving the mitochondrial membrane potential, Shao *et al.* [112] found that EMPA can regulate the mitochondrial fusion and division function. In addition, a study found that EMPA can restore mitochondrial DNA damage and improve mitochondrial biogenesis [40].

## 8 INHIBITION OF NHE1

The Na^+^/H^+^ exchanger (NHE) is a secondary active transporter responsible for the regulation of intracellular pH, Na^+^ concentration, and cell volume [113]. The NHE family has several different molecular isoforms. Among them, NHE1 is the major subtype in the heart [114] and a key target of numerous cardiovascular diseases, such as myocardial hypertrophy [115]. Increased NHE1 activity promotes the expression of genes that contribute to myocardial hypertrophy [114]. Increasing evidence suggests that increased NHE1 activity directly contributes to cardiac hypertrophy in experimental diabetic patients [116-118].

Previous studies on H9c2 cardiomyocytes have indicated that Ang II-mediated cardiomyocyte hypertrophy is accompanied by an increase in NHE1 protein expression [119, 120] and infecting H9c2 cardiomyocytes with active NHE1 leads to myocardial cell hypertrophy [121]. Inhibiting NHE1 may reduce cardiomyocyte injury, myocardial hypertrophy, fibrosis, and remodeling [122, 123].

Studies have elucidated that SGLT2 inhibitors have intrinsic NHE inhibition capacity [124, 125]. It has been shown that EMPA can directly inhibit NHE and play a role by decreasing myocardial [Na^+^]c and [Ca^2+^]c and augmenting [Ca^2+^]m [126]. Several research studies have similarly concluded that SGLT2 inhibitors reduced [Na^+^]c in the myocardium, which may be ascribed to their direct inhibition of NHE1 in the myocardium [108, 124] because the effect of EMPA was diminished, while cariporide, a selective NHE1 inhibitor, was used to pretreat cells [108].

Furthermore, SGLT2 inhibitors can reduce the upregulation of NHE in mouse cardiac fibroblasts by activating AMPK [127]. An increasing number of studies have demonstrated that SGLT2 inhibitors exert a direct induction-promoting effect on AMPK [128, 129], whereas AMPK can reduce NHE1 expression in mouse cardiac fibroblasts [127].

## 9 IMPROVEMENT OF INSULIN RESISTANCE

Insulin resistance is considered to be a cause of cardiac tissue changes in left ventricular hypertrophy, even in the absence of diabetes [130]. Insulin resistance may contribute to the expression of some myocardial cell hypertrophy genes [131], and high insulin levels, combined with insulin-like growth factor 1 receptor, may induce myocardial cell hypertrophy [132].

Many studies have demonstrated the role of SGLT2 inhibitors in improving insulin resistance and increasing insulin sensitivity. Brown *et al.* [14] observed a substantial decrease in insulin resistance due to dapagliflozin in DAPA-LVH. Another trial of patients with T2DM elucidated that EMPA ameliorated β-cell function and insulin sensitivity [133]. Mudaliar *et al.* also demonstrated that dapagliflozin treatment improved insulin sensitivity by reducing glycosylated hemoglobin and weight [134].

SGLT2 inhibitors promote the excretion of glucose from the kidney, exerting a hypoglycemic effect independent of the function of β-cells and the degree of insulin resistance. Interestingly, this continuous loss of glucose from the urine may lead to complex indirect metabolic effects. The increase of glycosuria induced by SGLT2 inhibitors can reduce the potential glucotoxicity of blood glucose, thus improving the insulin secretion β- cells and tissue insulin sensitivity [133, 135]. In addition, SGLT2 inhibitor EMPA also improves myocardial insulin sensitivity and glucose utilization by modulating diabetes-related increases in MAPKs and dysregulation of insulin receptor substrate 1 and Akt phosphorylation [136], thereby benefiting the improvement of myocardial hypertrophy and fibrosis.

## 10 IMPROVEMENT OF ENDOTHELIAL FUNCTION

Endothelial dysfunction is often accompanied by cardiac hypertrophy and fibrosis [137] and leads to increased passive tension in cardiomyocytes, cardiomyocyte hypertrophy, and impaired diastolic function [138]. Endothelial dysfunction related to left ventricular hypertrophy is at least partially attributable to Ang II-mediated nicotinamide adenine dinucleotide phosphate oxidase-2 activation [137]. A recent study confirmed that canagliflozin can reduce the expression of nicotinamide adenine dinucleotide phosphate oxidase subunits, such as nicotinamide adenine dinucleotide phosphate oxidase-2, and prevent endothelial dysfunction in diabetic ApoE -/- mice [139].

Several research studies have shown that in myocardial fibers from patients with heart failure, EMPA increases the phosphorylation level of myofilament regulatory proteins (myostatin) to reduce passive stiffness of cardiomyocytes and preserve nitric oxide signaling and cardiomyocyte function, thus preventing cardiac microvascular endothelial cell dysfunction [140]. This suggests that EMPA exerts a significant effect on the myocardium by ameliorating diastolic stiffness [140]. Moreover, SGLT2 inhibitors are thought to optimize endothelial function and vascular stiffness, which may improve hemodynamics by reducing afterload, thus improving myocardial hypertrophy [12]. Salim *et al.* [141] also found that ipragliflozin improved impaired endothelial function in streptozotocin-induced diabetic mice. In addition, dapagliflozin was also proven to ameliorate endothelial dysfunction in mice with T2DM in animal studies [142]. This evidence suggests that SGLT2 inhibitors can improve endothelial function in different states, thereby alleviating pathological myocardial hypertrophy.

## 11 UPREGULATION OF AUTOPHAGY

Autophagy is a cellular housekeeping procedure that is activated during nutrient shortage and inhibited during nutrient excess. Impaired organelles and possibly damaging cytoplasmic fragments are sent to lysosomes for degradation [143, 144]. Autophagy deals with malfunctioning mitochondria, thereby reducing oxidative stress. Impaired autophagy affects the normal degradation of damaged organelles and the normal folding of proteins, thus leading to increased oxidative stress in cardiac myocytes [145].

Akt and mTOR complex 1 (mTORC1) are serine/threonine protein kinases that are activated in response to nutrient overload, and the Akt/mTORC1 signaling pathway can inhibit autophagy. In contrast, sirtuin1 (SIRT1) is activated in response to nutrient deficiency and has the capacity to promote autophagy [146].

Upregulation of SIRT1 in myocardial cells can reduce oxidative stress and improve mitochondrial function [147]. SIRT1 can alleviate myocardial hypertrophy by mediating redox regulators as well as inflammatory body inhibitors [148]. Activation of SIRT1 can improve the fibrosis caused by pressure overload and prevent adverse ventricular remodeling after experimental infarction [149, 150]. Downstream effectors proliferator-activated receptor-gamma coactivator 1-alpha (PGC-1α) and fibroblast growth factor 21 (FGF21) partially mediated the action of SIRT1 and enhanced the action through AMPK [146].

T2DM is considered to be a state of overnutrition, along with downregulation of SIRT1/PGC-1α/FGF21 and AMPK and inhibition of autophagy [151]. Nevertheless, notably, autophagy increases in type 1 diabetic hearts [152], demonstrating that hyperinsulinemia in T2DM rather than hyperglycemia causes autophagy downregulation. Insulin may inhibit autophagy by inhibiting SIRT1 and activating the Akt/mTORC1 signaling pathway [153, 154].

Inhibition of the SIRT1/AMPK signaling pathway and autophagy is related to the pathogenesis of T2DM cardiomyopathy [152, 155]. On the contrary, experiments have shown that enhanced SIRT1/AMPK signaling pathways or autophagy can ameliorate impaired cardiac function [149, 150].

In other words, diabetes may lead to the activation of Akt/mTORC1 and the inhibition of SIRT1/PGC-1α/FGF21 through hyperinsulinemia, thereby inhibiting autophagic flux and promoting oxidative stress and mitochondrial dysfunction of diabetic myocardial cells, and thus promoting pathological cardiac hypertrophy.

The SGLT2 protein is a nutrient overload sensor, and its inhibition brings about increased glycosuria, resulting in a great deal of calorie loss *via* urine, which triggers a state of starvation and thus promotes the upregulation of SIRT1 and autophagy [78, 151, 156, 157]. In various tissues, such as the heart, SGLT2 inhibitors can activate SIRT1 as well as its downstream factors [79, 158-164]. Apart from affecting SIRT1/PGC-1α/FGF21, these agents can inhibit Akt/mTOR [79] and stimulate AMPK [165, 166].

The synergy of these signals may underlie the promotion of autophagic flux by SGLT2 inhibitors in various organs [40, 167-170]. This may also be the reason why these agents alleviate oxidative stress, improve mitochondrial dysfunction, and inhibit pro-inflammatory signaling [35, 40, 61, 168]. Enhanced autophagy also explains why SGLT2 inhibitors can improve myocardial hypertrophy and fibrosis and the course of experimental cardiomyopathy [17, 35, 171].

## CONCLUSION AND PERSPECTIVES

Hypertrophy includes physiological hypertrophy as well as pathological hypertrophy. Physiological hypertrophy has normal or enhanced systolic function, which contributes to heart function, and the heart structure remains normal [172], whereas pathological hypertrophy is related to myocardial fibrosis and structural remodeling [4], which usually increases the risk of adverse cardiovascular events [5].

SGLT2 inhibitors were originally developed as hypoglycemic agents with a unique hypoglycemic approach that differs from other hypoglycemic drugs. They reduce blood sugar levels by increasing urine glucose excretion without increasing the risk of hypoglycemia. In the process of drug application, it has been gradually found that it can improve pathological myocardial hypertrophy. The recent EMPA-HEART Cardiolink-6 trial found that the left ventricular mass, as measured by body surface area, decreased significantly after 6 months of EMPA treatment in patients with T2DM and coronary artery disease [13]. A randomized controlled experiment (DAPA-LVH) [14] proposed by Brown *et al.* further demonstrated that the SGLT2 inhibitor, dapagliflozin, can lead to reverse remodeling and changes of left ventricular structure in T2DM patients with left ventricular hypertrophy [14]. Moreover, four weeks of EMPA treatment ameliorated myocardial hypertrophy and myocardial fibrosis induced by chronic cortisol in mice [16]. A decrease in left ventricular mass and septal thickness and improvement of cardiomyocyte hypertrophy and ventricular fibrosis were found in rats with non-diabetic cardiomyopathy treated with the SGLT2 inhibitor, ipragliflozin [17].

In this review, we comprehensively analyzed the role of SGLT2 inhibitors in attenuating pathological cardiac hypertrophy. Due to its unique hypoglycemic mode, SGLT2 inhibitors can provide stable and long-lasting hypoglycemic effects at any stage of the natural course of T2DM, thereby alleviating pathological cardiac hypertrophy caused by diabetes. More importantly, however, cardiomyocyte hypertrophy and myocardial fibrosis can be ameliorated by the application of SGLT2 inhibitors, even in the absence of diabetes. The above experimental studies have shown that SGLT2 inhibitors have many mechanisms of reducing pathological myocardial hypertrophy that are not related to hypoglycemic effects, which are summarized in Fig. (**2**), including reducing myocardial inflammation and oxidative stress, reducing the volume of epicardial adipose tissue, improving myocardial mitochondrial function, improving myocardial insulin resistance, inhibiting cardiomyocyte NHE1, and preventing cardiac microvascular endothelial cell dysfunction. In addition, the upregulation of autophagy caused by SGLT2 inhibitors is also a mechanism worth exploring. SGLT2 inhibitors can promote autophagic flux in different organs, which may underlie the attenuation of oxidative stress, improvement of mitochondrial dysfunction, and suppression of pro-inflammatory signaling.

In summary, SGLT2 inhibitors have broad therapeutic prospects and are undoubtedly beneficial for T2DM patients with myocardial hypertrophy. However, according to our review, we believe that the application of SGLT2 inhibitors in pathological myocardial hypertrophy should not be limited to patients with T2DM. For patients without T2DM, the application of SGLT2 inhibitors can also reduce myocardial hypertrophy and even reverse ventricular remodeling, reducing the risk of cardiovascular events. Although some progress has been made, current studies are limited to animal experiments, and the clinical application of SGLT2 inhibitors in non-diabetic myocardial hypertrophy remains to be further explored.

## Figures and Tables

**Table 1:** SGLT2 inhibitors-induced changes in pathological myocardial hypertrophy.

**-**	**Subjects**	**SGLT2 Inhibitor**	**Dosage**	**Observation** **Period**	**Effects of Treatment**	**References**
Animal experiments	SHRcp rats	Empagliflozin	A standard diet containing 0.03% empagliflozin	10 weeks	Empagliflozin ameliorated cardiac hypertrophy and fibrosis.	Kusaka *et al.* (2016)
-	Male C57BL/6J mice	Empagliflozin	10 mg/kg/day	4 weeks	Left ventricular hypertrophy and cardiac dysfunction were improved significantly, phosphorylated STAT3 and TLR4 were alleviated, and macrophage infiltration in the myocardium was inhibited.	Zhang *et al.* (2020)
-	Non-diabetic DS/obese rats	Ipragliflozin	A standard diet containing 0.01% empagliflozin	8 weeks	Ipragliflozin reduced left ventricular mass and intraventricular septal thickness and ameliorated hypertrophy of cardiomyocytes and left ventricular fibrosis.	Takasu *et al.* (2019)
-	diabetic KK-Ay mice	Empagliflozin	10 mg/kg/day	8 weeks	Empagliflozin improved diabetic myocardial structure and function, suppressed oxidative stress and fibrosis through inhibition of the transforming growth factor β/Smad pathway and activation of Nrf2/ARE signaling	Li *et al.* (2019)
-	C57BL/6J mice	Ertugliflozin	225 mg/kg chow diet	10 weeks	Ertugliflozin improved left ventricular function and reduced myocardial fibrosis.	Moellmann *et al.* (2022)
-	HFD-fed mice	Dapagliflozin	1 mg/kg/day	2 months	Dapagliflozin alleviated HFD-induced cardiac dysfunction and cardiac aberrant remodeling.	Lin *et al.* (2022)
-	SD rats	Dapagliflozin	5 mg/kg/day	4 weeks	Myocardial hypertrophy, fibrosis and increased collagen synthesis caused by Ang II infusion were significantly inhibited by Dapagliflozin pretreatment.	Zhang *et al.* (2021)
-	db/db mice	Empagliflozin	10 mg/kg/day	5 weeks	Empagliflozin improved eccentric left ventricular hypertrophy, indicated by a reduction in cardiomyocyte cross-sectional area.	Habibi *et al.* (2017)
-	C57BL/6J mice	Empagliflozin	10 mg/kg/day	8 weeks	Empagliflozin improved myocardial hypertrophy/fibrosis and cardiac function and reduced cardiac fat accumulation and mitochondrial injury.	Sun *et al.* (2020)
-	C57BL/6J mice	Dapagliflozin	1 mg/kg/day	4 weeks	Dapagliflozin treatment reduced myocardial hypertrophy, myocardial interstitial and perivascular fibrosis.	Shi *et al.* (2019)
Clinical studies	T2D and CAD	Empagliflozin	10 mg/day	6 months	Empagliflozin was associated with a significant reduction in left ventricular mass indexed to body surface area after 6 months.	Verma *et al.* (2019)
-	T2D	Dapagliflozin	10 mg/day	12 months	Dapagliflozin treatment significantly reduced left ventricular mass in people with type 2 diabetes and left ventricular hypertrophy.	Brown *et al.* (2020)

## LIST OF ABBREVIATIONS

Akt: Protein Kinase B
AMPK: Adenosine-monophosphate-activated Protein Kinase
Ang II: Angiotensin II
EAT: Epicardial Adipose Tissue
EMPA: Empagliflozin
FGF21: Fibroblast Growth Factor 21
MAPK: Mitogen-activated Protein Kinase
mTOR: Mammalian Target of Rapamycin
mTORC1: Mammalian Target of Rapamycin Complex 1
NHE1: Sodium-hydrogen Exchanger Isoform-1
NLRP3: Nucleotide Oligomerization Domain-like Receptor Pyrin Domain Containing 3
PGC-1α: Proliferator-activated Receptor-gamma Coactivator 1-alpha
PKC: Protein Kinase C
PPARGC1A: Peroxisome Proliferator-activated Receptor-gamma Coactivator-α
ROS: Reactive Oxygen Species
SGK1: Serum- and Glucocorticoid-responsive Kinase-1
SGLT2: Sodium-glucose Cotransport Protein 2
SIRT1: Sirtuin1
STAT3: Signal Transducer and Activator of Transcription 3
TAC: Transverse Aortic Constriction
TGFβ1: Transforming Growth Factor-β1
TLR4: Toll-like Receptor 4
TNFRSF12a: Tumor Necrosis Factor Receptor Superfamily Member 12a
TNFSF12: Tumor Necrosis Factor Superfamily Member 12
